# Impact of occupation on survival of esophageal squamous cell carcinoma patients following esophagectomy: a long-term survival analysis

**DOI:** 10.1186/s12876-025-03890-3

**Published:** 2025-04-24

**Authors:** Kexun Li, Simiao Lu, Jie Mao, Huan Zhang, Kangning Wang, Guangyuan Liu, Qifeng Wang, Yongtao Han, Lin Peng, Xuefeng Leng

**Affiliations:** 1 Department of Thoracic Surgery, Sichuan Clinical Research Center for Cancer, Sichuan Cancer Hospital & Institute, School of Medicine, Sichuan Cancer Center, Affiliated Cancer Hospital of University of Electronic Science and Technology of China (Sichuan Cancer Hospital), Chengdu, People’s Republic of China; 2 Department of Thoracic Surgery I, Third Affiliated Hospital of Kunming Medical University (Yunnan Cancer Hospital, Yunnan Cancer Center), Kunming, China; 3 Department of Radiation Oncology, School of Medicine, Sichuan Cancer Hospital & Institute, University of Electronic Science and Technology of China (UESTC), Chengdu, China; 4 School of Public Health, Chongqing Medical University, Chongqing, China

**Keywords:** Esophageal Squamous Cell Carcinoma, Occupation, Esophagectomy, Survival Analysis, Risk Factors

## Abstract

**Background:**

Esophageal cancer (EC), particularly esophageal squamous cell carcinoma (ESCC), is a major global health issue with high incidence and mortality rates in Asia. This study examines the impact of occupational background on the long-term survival of ESCC patients following esophagectomy.

**Methods:**

Data were obtained from the Sichuan Cancer Hospital & Institute Esophageal Cancer Case Management Database (SCCH-ECCM Database), focusing on patients with ESCC who underwent esophagectomy between 2010 and 2017. Patients were classified into four occupational groups: Farmer, Civil Servant, Teacher, and Factory Worker. The primary outcome measured was overall survival (OS), which was analyzed using Kaplan–Meier survival curves, Cox proportional hazards models, and restricted mean survival time (RMST). To account for potential confounding factors, propensity score matching (PSM) was employed.

**Results:**

Among the cohort, 67.5% were Farmers, 9.0% Civil Servants, 1.9% Teachers, and 21.6% Factory Workers. The median follow-up was 72.2 months, with a median OS of 49.8 months. One-, three-, and five-year OS rates varied slightly by occupation, with Factory Workers displaying the highest one-year survival rate at 91%. Significant survival differences were noted between Farmers and Civil Servants (HR: 1.291; 95% CI: 1.030 − 1.618; *P* = 0.027),the significance persisted even after PSM (HR: 1.376; 95% CI: 1.004 − 1.885; *P* = 0.047). Civil Servants, who presented with more advanced disease stages, had the lowest crude RMST, aligning more closely with other groups after adjustment.

**Conclusions:**

The results revealed that ESCC patients categorized as Civil Servants exhibited a poorer prognosis compared to those classified as Farmers.

## Introduction

Esophageal cancer (EC) is a significant global health concern, marked by notable regional variations in incidence and mortality. It is particularly prevalent in parts of Asia, including China and Japan, where the rates remain alarmingly high. In 2020, there were approximately 604,000 new cases of esophageal cancer worldwide, leading to 544,000 deaths [1, 2]. The disease primarily manifests as two main histological subtypes: esophageal squamous cell carcinoma (ESCC) and esophageal adenocarcinoma (EAC), with ESCC being the dominant subtype. These subtypes differ in their risk factors: ESCC is predominantly associated with alcohol consumption and tobacco smoking, whereas EAC is more closely linked to obesity and gastroesophageal reflux disease. As the global population ages, the incidence of esophageal cancer among elderly patients is increasing, presenting unique challenges in management and treatment outcomes [3–6].

Current treatment for esophageal cancer is centered around surgery, particularly for patients with locally advanced disease. This often involves a multimodal approach that includes neoadjuvant chemoradiotherapy, chemotherapy, or chemoradiation therapy before surgery [7–10]. Postoperative pathological results are then used to determine the necessity for additional adjuvant chemotherapy or immunotherapy [11, 12].

Recent research has highlighted that environmental factors can significantly influence the development and progression of cancer [13–16]. Patients from diverse occupational backgrounds often experience varying environmental exposures, which can affect their overall health and cancer outcomes. For instance, individuals engaged in physical labor may possess different physical fitness levels compared to those involved primarily in intellectual work. Additionally, economic disparities associated with various occupations can further impact health outcomes and access to care. This study aims to explore the long-term overall survival (OS) of patients with differing occupational backgrounds who undergo esophagectomy. By examining these differences, the research seeks to provide insights into the impact of occupation on the long-term OS of ESCC patients following esophagectomy.

## Materials and methods

### Patients characteristics

**Fig. 1:**
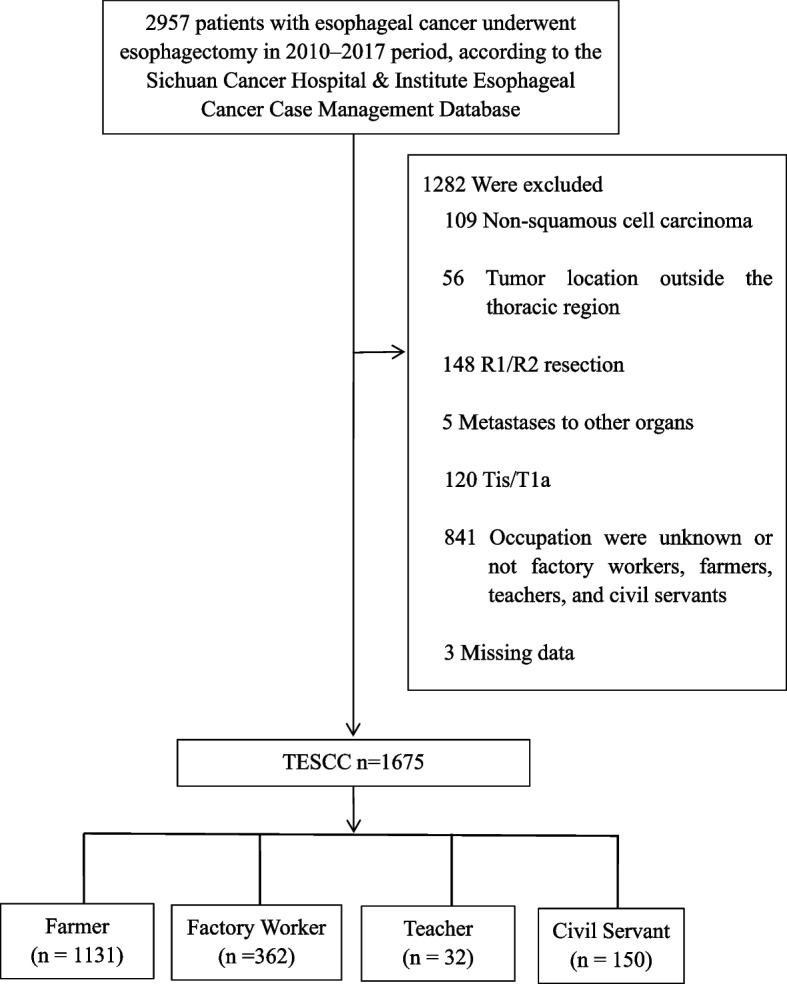
CONSORT diagram showing patient selection. TESCC, thoracic esophageal squamous cell carcinoma

This retrospective cohort study utilized data from the Sichuan Cancer Hospital & Institute Esophageal Cancer Case Management Database (SCCH-ECCM Database), focusing on patients diagnosed with esophageal squamous cell carcinoma (ESCC) who underwent esophagectomy between January 2010 and December 2017. A total of 2,957 patients were initially considered. Patients were included if they had thoracic ESCC without distant metastasis. Out of the initial cohort, 1,675 patients met the inclusion criteria and were divided into four occupational groups for analysis: Farmer, Civil Servant, Teacher, and Factory Worker. Exclusion criteria led to the removal of 1,282 patients for the following reasons: (1) non-squamous cell carcinoma, (2) tumors located outside the thoracic region, (3) underwent R1/R2 resection indicating incomplete tumor removal, (4) metastases to other organs, (5) pTis/T1a stage, (6) unknown or inapplicable occupations, and (7) had missing data (Fig. 1).

### Grouping and outcome measures

The study groups were categorized based on patients'occupational backgrounds. Data collected included demographic details, clinical and pathological characteristics, and postoperative outcomes. Disease staging adhered to the 8 th edition TNM classification of the UICC/AJCC. Pathological findings were reviewed by two pathologists and validated by a third to ensure accuracy. The primary outcome measure was OS, assessed from the date of surgery until death or last follow-up.

### Statistical analyses

Descriptive statistics, including means and standard deviations for continuous variables and counts and percentages for categorical variables, were used to summarize patient characteristics. Kaplan–Meier survival curves were generated for OS, with group differences analyzed using the log-rank test. To account for potential confounding factors, propensity score matching (PSM) was employed. Cox proportional hazards regression models calculated hazard ratios (HRs) and 95% confidence intervals (CIs) to identify independent risk factors for OS. Univariate Cox regression analyses were conducted, followed by multivariate analyses adjusting for potential confounders such as age, sex, and tumor stage, in our multivariate analysis, we included variables that showed a significant impact on survival in the univariate analysis (*p* < 0.05). Comparisons between groups were made using the log-rank test. Restricted mean survival time (RMST) estimates along with 95% CIs were calculated for both groups based on Kaplan–Meier estimates. Statistical analyses were performed using RStudio software running R version 4.3.0, with a significance level set at *p* < 0.05.

## Results

### Patients characteristics

From the initial cohort of 2,957 ESCC patients, 1,675 were included in the final analysis. The distribution of patients according to occupation was as follows: 1131 Farmers (67.5%), 150 Civil Servants (9.0%), 32 Teachers (1.9%), and 362 Factory Workers (21.6%). The majority of the patients were male, comprising 1,384 individuals or 82.6% of the cohort, while females accounted for 291 individuals or 17.4%. Regarding age distribution, 81 patients (4.8%) were aged 75 and above, while 1594 patients (95.2%) were younger than 75. In terms of pathological staging, approximately 72.5% of the patients were classified as T3 or T4, indicating advanced tumor depth. Furthermore, 57.6% of the patients had confirmed lymph node (LN) metastasis post-esophagectomy. A significant proportion of patients, 980 (58.5%), were staged beyond stage III pathologically. Adjuvant therapy post-surgery was administered to more than half of the patients (Table 1).

**Table 1:** Demographic characteristics of the patients

Variables	Total (*n* = 1,675)	Farmer (*n* = 1,131)	Civil Servant (*n* = 150)	Teacher (*n* = 32)	Factory Worker (*n* = 362)
Sex					
Male	1,384 (82.6%)	891 (78.8%)	141 (94.0%)	29 (90.6%)	323 (89.2%)
Female	291 (17.4%)	240 (21.2%)	9 (6.0%)	3 (9.4%)	39 (10.8%)
Age, years					
Median (range)		61 (34–85)	63.5 (40–82)	65 (35–78)	61 (38–84)
< 75	1,594 (95.2%)	1,083 (95.8%)	136 (90.7%)	30 (93.8%)	345 (95.3%)
≥ 75	81 (4.8%)	48 (4.2%)	14 (9.3%)	2 (6.2%)	17 (4.7%)
KPS score					
≤ 80	631 (37.7%)	396 (35.0%)	47 (31.3%)	6 (18.7%)	182 (50.3%)
> 80	1,044 (62.3%)	735 (65.0%)	103 (68.7%)	26 (81.3%)	180 (49.7%)
Smoking	696 (41.6%)	404 (35.7%)	69 (46.0%)	11 (34.4%)	212 (58.6%)
Alcohol	715 (42.7%)	421 (37.2%)	75 (50.0%)	9 (28.1%)	210 (58.0%)
Pathological differentiation grade					
Moderate or Well G1 - 2	987 (58.9%)	668 (59.1%)	94 (62.7%)	17 (53.1%)	208 (57.5%)
Poor or undifferentiated G3 - 4	688 (41.1%)	463 (40.9%)	56 (37.3%)	15 (46.9%)	154 (42.5%)
Tumor location					
Upper	424 (25.3%)	310 (27.4%)	31 (20.7%)	10 (31.2%)	73 (20.2%)
Middle	884 (52.8%)	609 (53.8%)	84 (56.0%)	15 (46.9%)	176 (48.6%)
Lower	367 (21.9%)	212 (18.8%)	35 (23.3%)	7 (21.9%)	113 (31.2%)
Lymphovascular invasion					
Yes	292 (17.4%)	176 (15.6%)	32 (21.3%)	3 (9.4%)	81 (22.4%)
No	1,383 (82.6%)	955 (84.4%)	118 (78.7%)	29 (90.6%)	281 (77.6%)
Nerve invasion					
Yes	324 (19.3%)	217 (19.2%)	35 (23.3%)	3 (9.4%)	69 (19.1%)
No	1,351 (80.7%)	914 (80.8%)	115 (76.7%)	29 (90.6%)	293 (80.9%)
Pathological T stage					
T1b	128 (7.6%)	94 (8.3%)	8 (5.3%)	5 (15.6%)	21 (5.8%)
T2	334 (19.9%)	219 (19.4%)	25 (16.7%)	7 (21.9%)	83 (22.9%)
T3	1,068 (63.8%)	722 (63.8%)	102 (68.0%)	16 (50.0%)	228 (63.0%)
T4	145 (8.7%)	96 (8.5%)	15 (10.0%)	4 (12.5%)	30 (8.3%)
N stage					
N0	710 (42.4%)	507 (44.8%)	55 (36.7%)	12 (37.5%)	136 (37.6%)
N1	499 (29.8%)	328 (29.0%)	40 (26.7%)	11 (34.4%)	120 (33.1%)
N2	303 (18.1%)	194 (17.2%)	41 (27.3%)	6 (18.7%)	62 (17.1%)
N3	163 (9.7%)	102 (9.0%)	14 (9.3%)	3 (9.4%)	44 (12.2%)
8 th TNM Stage					
I	125 (7.5%)	85 (7.5%)	11 (7.3%)	3 (9.4%)	26 (7.2%)
II	570 (34.0%)	413 (36.5%)	38 (25.3%)	10 (31.2%)	109 (30.1%)
III	764 (45.6%)	489 (43.2%)	81 (54.0%)	16 (50.0%)	178 (49.2%)
IV	216 (12.9%)	144 (12.7%)	20 (13.3%)	3 (9.4%)	49 (13.5%)
Thoracic surgery					
MIE	695 (41.5%)	515 (45.5%)	63 (42.0%)	12 (37.5%)	105 (29.0%)
OE	980 (58.5%)	616 (54.5%)	87 (58.0%)	20 (62.5%)	257 (71.0%)
Abdominal surgery					
MIE	521 (31.1%)	401 (35.4%)	47 (31.3%)	7 (21.9%)	66 (18.2%)
OE	1,152 (68.8%)	728 (64.4%)	103 (68.7%)	25 (78.1%)	296 (81.8%)
No	2 (0.1%)	2 (0.2%)	0 (0.0%)	0 (0.0%)	0 (0.0%)
Clinical treatment method					
Surgery alone	797 (47.6%)	602 (53.2%)	78(52.0%)	10 (31.3%)	107(29.6%)
Preoperative CT or RT/CRT + surgery	32(1.9%)	17(1.5%)	7(4.7%)	1(3.1%)	7(1.9%)
Surgery + Postoperative CT or RT/CRT	846 (50.5%)	512 (45.3%)	65(43.3%)	21 (65.6%)	248(68.5%)

*CT* Chemotherapy, *CRT* Chemoradiotherapy, *MIE* Minimally invasive esophagectomy, *OE* Open esophagectomy, *RT* Radiotherapy, *TNM* Tumor, node, metastasis

### Survival outcomes

**Fig. 2:**
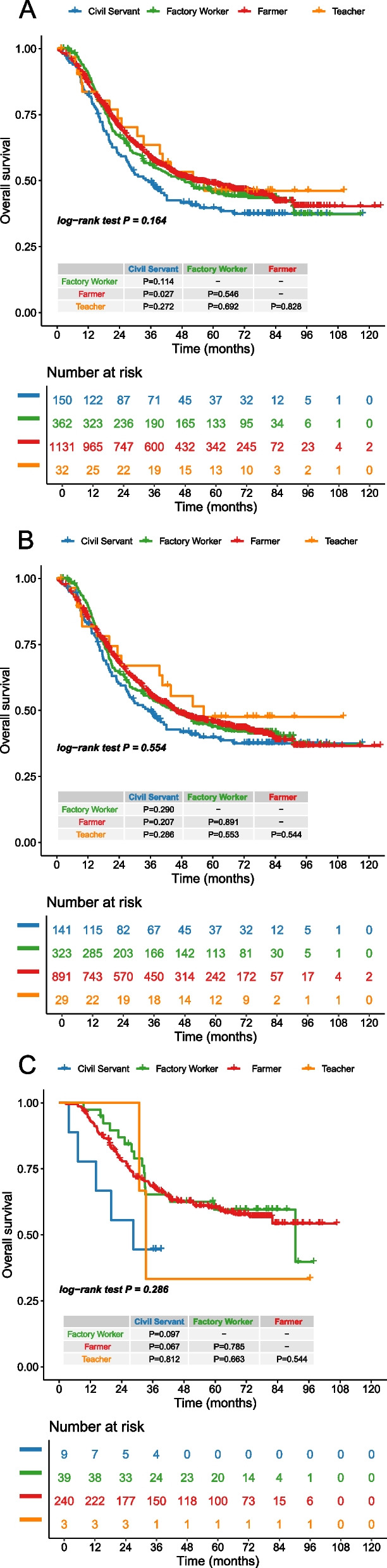
**A** The overall survival curve of patients in the 4 groups; (**B**) The overall survival curve of male in the 4 groups; (**C**) The overall survival curve of female in the 4 groups

**Fig. 3:**
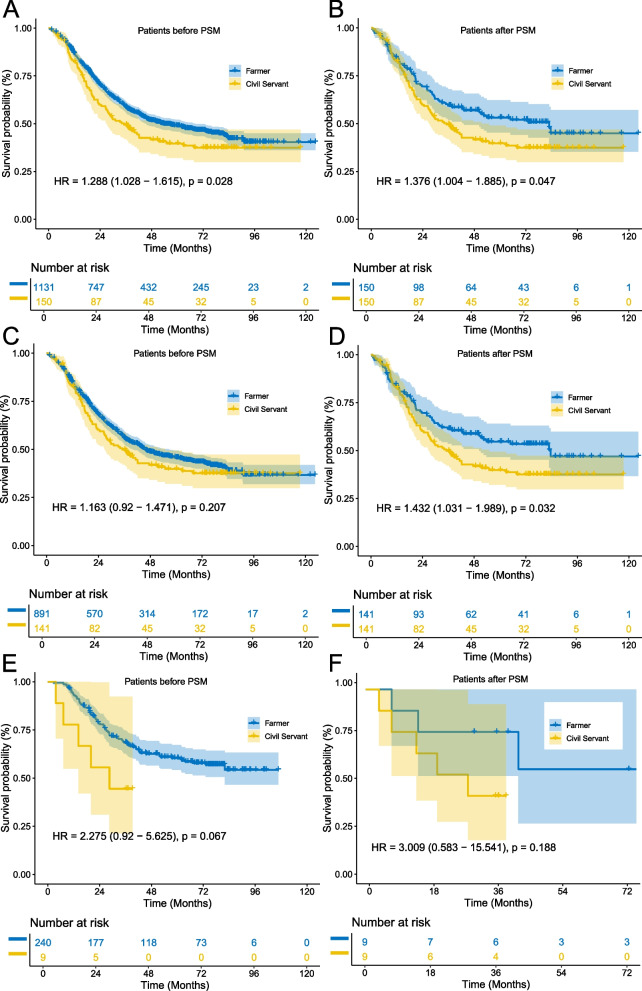
**A** The overall survival curve of Farmers and Civil Servants before PSM; (**B**) The overall survival curve of Farmers and Civil Servants after PSM. **C** The overall survival curve of Farmers and Civil Servants before PSM in male; (**D**) The overall survival curve of Farmers and Civil Servants after PSM in male. **E** The overall survival curve of Farmers and Civil Servants before PSM in female; (**F**) The overall survival curve of Farmers and Civil Servants after PSM in female

The median follow-up time for the included patients was 72.2 months, with a median OS time of 49.8 months. The OS rates for the entire cohort were 87% at one year, 57% at three years, and 47% at five years. When broken down by occupational groups, the OS rates varied slightly. Farmers had a one-year OS rate of 87%, a three-year OS rate of 59%, and a five-year OS rate of 49%. Civil Servants experienced a one-year OS rate of 83%, a three-year OS rate of 50%, and a five-year OS rate of 40%. Teachers showed a one-year OS rate of 84%, a three-year OS rate of 64%, and a five-year OS rate of 46%. Factory Workers had the highest one-year OS rate at 91%, with a three-year OS rate of 56% and a five-year OS rate of 46%. OS analysis presented in Fig. 2 indicated no statistically significant differences in OS among the various occupational groups, with the exception of a significant difference between Farmers and Civil Servants (HR: 1.288; 95% CI: 1.028 − 1.615; *P* = 0.027; Figs. 2A and 3A), in the male and female subgroups, there were no statistically significant differences among all four groups. Due to the observed differences in TNM stage between Farmers and Civil Servants, with Civil Servants exhibiting a higher proportion of patients in the locally advanced stage (Table 2), we conducted a PSM analysis. The results indicated a statistically significant difference in OS between the two groups after PSM (HR: 1.376; 95% CI: 1.004 − 1.885; *P* = 0.047; Fig. 3B). This finding suggests that, even after adjusting for differences in TNM stage, Civil Servants continue to demonstrate poorer survival outcomes compared to Farmers. In the male subgroup, no significant difference was observed between Farmers and Civil Servants (HR: 1.163; 95% CI: 0.920 − 1.471; *P* = 0.207; Figs. 2B and 3C). However, after PSM, a significant difference was noted (HR: 1.432; 95% CI: 1.031 − 1.989; *P* = 0.032; Fig. 3D). In the female subgroup, no significant differences were found between Farmers and Civil Servants both before and after PSM (Fig. 3E and F).

**Table 2:** Demographic characteristics of the 2 groups

Characteristic	Before PSM		*P* value	After PSM		*P* value
	Farmer (*n* = 1131)	Civil Servant (*n* = 150)		Farmer (*n* = 150)	Civil Servant (*n* = 150)	
Sex			< 0.001			0.156
Male	891 (78.8%)	141 (94.0%)		146 (97.3%)	141 (94.0%)	
Female	240 (21.2%)	9 (6.0%)		4 (2.7%)	9 (6.0%)	
Age, years			0.006			1.0
Median (range)	61 (34–85)	63.5 (40–82)		62 (40–80)	63.5 (40–82)	
< 75	1,083 (95.8%)	136 (90.7%)		136 (90.7%)	136 (90.7%)	
≥ 75	48 (4.2%)	14 (9.3%)		14 (9.3%)	14 (9.3%)	
KPS score			0.373			0.447
≤ 80	396 (35.0%)	47 (31.3%)		41 (27.3%)	47 (31.3%)	
> 80	735 (65.0%)	103 (68.7%)		109 (72.7%)	103 (68.7%)	
Smoking	404 (35.7%)	69 (46.0%)	0.014	71 (47.3%)	69 (46.0%)	0.817
Alcohol	421 (37.2%)	75 (50.0%)	0.003	80 (53.3%)	75 (50.0%)	0.563
Pathological differentiation grade			0.398			0.630
Moderate or Well G1 - 2	668 (59.1%)	94 (62.7%)		98 (65.3%)	94 (62.7%)	
Poor or undifferentiated G3 - 4	463 (40.9%)	56 (37.3%)		52 (34.7%)	56 (37.3%)	
Tumor location			0.148			0.672
Upper	310 (27.4%)	31 (20.7%)		32 (21.3%)	31 (20.7%)	
Middle	609 (53.8%)	84 (56.0%)		77 (51.3%)	84 (56.0%)	
Lower	212 (18.8%)	35 (23.3%)		41 (27.3%)	35 (23.3%)	
Lymphovascular invasion			0.072			0.667
Yes	176 (15.6%)	32 (21.3%)		29 (19.3%)	32 (21.3%)	
No	955 (84.4%)	118 (78.7%)		121 (80.7%)	118 (78.7%)	
Nerve invasion			0.230			0.667
Yes	217 (19.2%)	35 (23.3%)		32 (21.3%)	35 (23.3%)	
No	914 (80.8%)	115 (76.7%)		118 (78.7%)	115 (76.7%)	
Pathological T stage			0.442			0.896
T1b	94 (8.3%)	8 (5.3%)		7 (4.7%)	8 (5.3%)	
T2	219 (19.4%)	25 (16.7%)		30 (20.0%)	25 (16.7%)	
T3	722 (63.8%)	102 (68.0%)		98 (65.3%)	102 (68.0%)	
T4	96 (8.5%)	15 (10.0%)		15 (10.0%)	15 (10.0%)	
N stage			0.021			0.550
N0	507 (44.8%)	55 (36.7%)		65 (43.3%)	55 (36.7%)	
N1	328 (29.0%)	40 (26.7%)		41 (27.3%)	40 (26.7%)	
N2	194 (17.2%)	41 (27.3%)		32 (21.3%)	41 (27.3%)	
N3	102 (9.0%)	14 (9.3%)		12 (8.0%)	14 (9.3%)	
8 th TNM Stage			0.042			0.623
I	85 (7.5%)	11 (7.3%)		9 (6.0%)	11 (7.3%)	
II	413 (36.5%)	38 (25.3%)		48 (32.0%)	38 (25.3%)	
III	489 (43.2%)	81 (54.0%)		76 (50.7%)	81 (54.0%)	
IV	144 (12.7%)	20 (13.3%)		17 (11.3%)	20 (13.3%)	
Thoracic surgery			0.414			0.726
MIE	515 (45.5%)	63 (42.0%)		66 (44.0%)	63 (42.0%)	
OE	616 (54.5%)	87 (58.0%)		84 (56.0%)	87 (58.0%)	
Abdominal surgery			0.526			0.901
MIE	401 (35.4%)	47 (31.3%)		46 (30.7%)	47 (31.3%)	
OE	728 (64.4%)	103 (68.7%)		104 (69.3%)	103 (68.7%)	
No	2 (0.2%)	0 (0.0%)		0 (0.0%)	0 (0.0%)	
Clinical treatment method			0.027			0.841
Surgery alone	602 (53.2%)	78(52.0%)		79 (52.7%)	78(52.0%)	
Preoperative CT or RT/CRT + surgery	17(1.5%)	7(4.7%)		5 (3.3%)	7(4.7%)	
Surgery + Postoperative CT or RT/CRT	512 (45.3%)	65(43.3%)		66 (44.0%)	65(43.3%)	

*KPS* Karnofsky Performance Status, *PSM* Propensity score matching, *TNM* Tumor, node, metastasis

### Restricted mean survival time

**Fig. 4:**
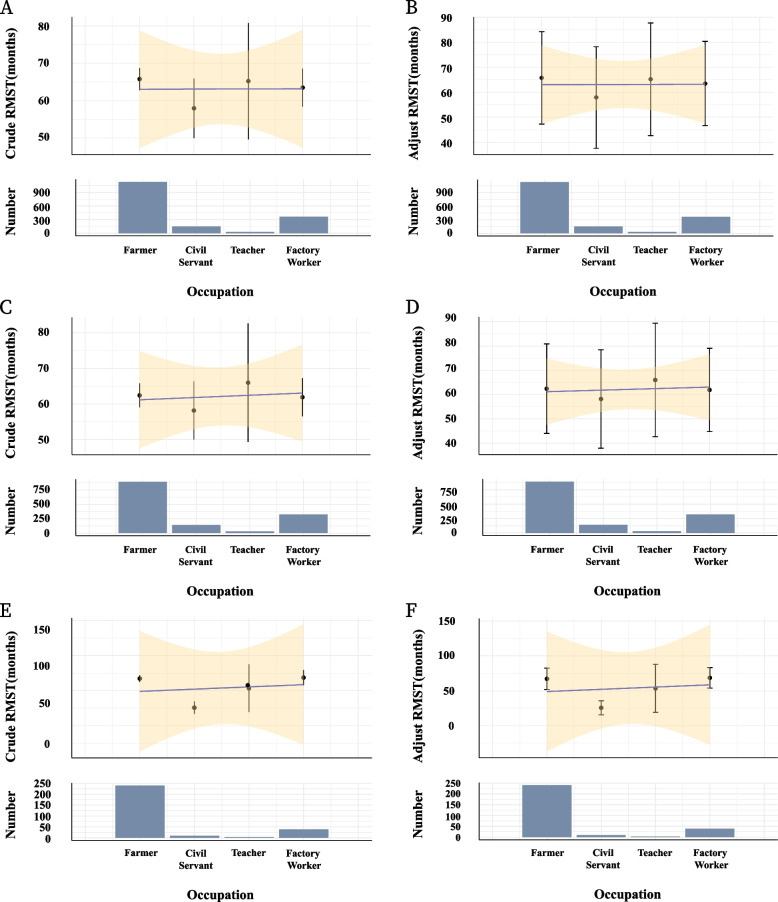
Restricted mean survival time (RMST) estimates patients. **A** Crude RMST estimates different patients; (**B**) Adjust RMST estimates different patients; (**C**) Crude RMST estimates different male patients; (**D**) Adjust RMST estimates different male patients; (**E**) Crude RMST estimates different female patients; (**F**) Adjust RMST estimates different female patients

The advanced staging observed in the Civil Servant group compared to the Farmer group and other occupational groups, particularly in terms of pathological T and N stages (TNM stage), we prompted further analysis using RMST. In the overall cohort, the Crude RMST for Farmers was 65.77 months (95% CI: 62.78–68.76), with an Adjusted RMST of 65.75 months (95% CI: 47.27–84.24) after accounting for potential confounders via propensity score matching (PSM). In contrast, Civil Servants exhibited a markedly lower Crude RMST of 57.93 months (95% CI: 49.95–65.90) and an Adjusted RMST of 57.94 months (95% CI: 37.68–78.21). Teachers recorded a Crude RMST of 65.22 months (95% CI: 49.65–80.79) and an Adjusted RMST of 65.23 months (95% CI: 42.73–87.74), while Factory Workers had a Crude RMST of 63.48 months (95% CI: 58.40–68.56) and an Adjusted RMST of 63.49 months (95% CI: 46.66–80.32). Across all occupational groups, Civil Servants consistently demonstrated the lowest RMST values in both crude and adjusted analyses (Fig. 4A and B). In the male subgroup, the Crude RMST for Farmers was 62.44 months (95% CI: 59.08–65.80), with an Adjusted RMST of 62.43 months (95% CI: 44.01–80.85). Civil Servants, however, had a lower Crude RMST of 58.19 months (95% CI: 50.02–66.37) and an Adjusted RMST of 58.21 months (95% CI: 37.94–78.48). Teachers showed a Crude RMST of 66.02 months (95% CI: 49.38–82.65) and an Adjusted RMST of 66.03 months (95% CI: 42.67–89.38), while Factory Workers had a Crude RMST of 61.93 months (95% CI: 56.58–67.28) and an Adjusted RMST of 61.94 months (95% CI: 44.77–79.10). Similar to the overall cohort, Civil Servants in the male subgroup exhibited the lowest RMST curves, both before and after adjustment for confounders (Fig. 4C and D). This finding aligns with the overall pattern, reinforcing the association between the Civil Servant occupation and reduced survival time among male ESCC patients. For the female subgroup, the Crude RMST for Farmers was 67.32 months (95% CI: 62.54–72.09), with an Adjusted RMST of 67.31 months (95% CI: 51.96–82.65). In stark contrast, Civil Servants had a significantly lower Crude RMST of 25.59 months (95% CI: 16.54–34.65) and an Adjusted RMST of 25.59 months (95% CI: 15.38–35.80). Teachers recorded a Crude RMST of 53.53 months (95% CI: 19.19–87.88) and an Adjusted RMST of 53.56 months (95% CI: 18.93–88.19), while Factory Workers had a Crude RMST of 68.70 months (95% CI: 57.76–79.64) and an Adjusted RMST of 68.71 months (95% CI: 53.93–83.49). Notably, Civil Servants in the female subgroup exhibited the lowest RMST values by a substantial margin, with the gap persisting after adjustment (Fig. 4E and F). The wider confidence intervals in this subgroup suggest a smaller sample size, which may limit statistical power, yet the trend of poorer survival among Civil Servants remains evident.

### Risk factors

**Fig. 5:**
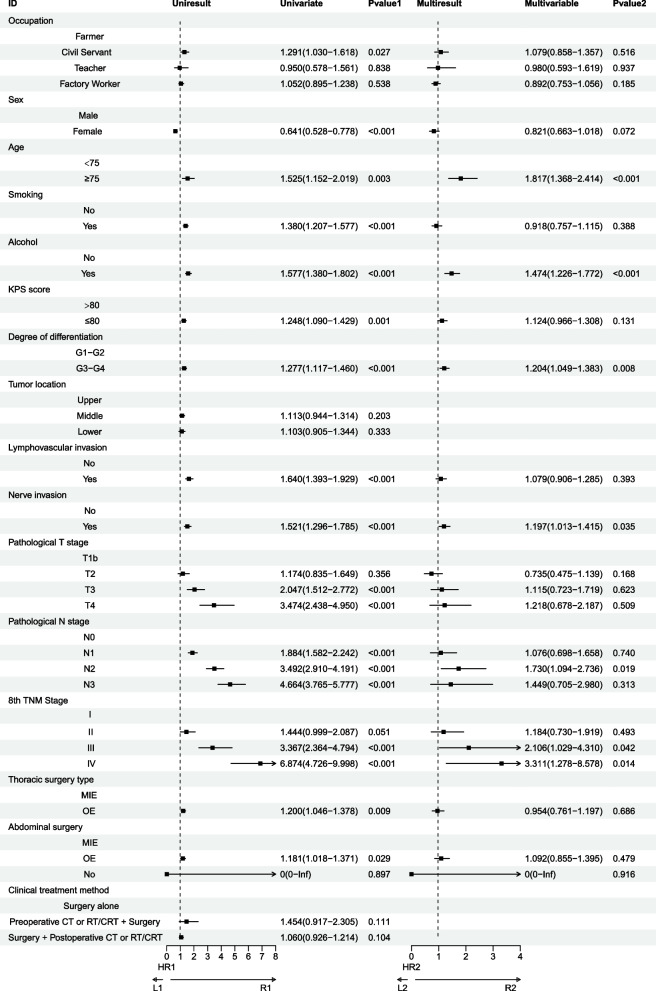
Univariate and multivariate Cox regression analyses for factors affecting patient survival. CI: confidence interval; CT: chemotherapy; CRT: chemoradiotherapy; HR: hazard ratio; LN: lymph node; MIE: minimally invasive esophagectomy; OE: open esophagectomy; RT: radiotherapy; TNM: tumor, node, metastasis

In the univariate analysis, several factors were identified as significantly impacting patient survival. Compared to Farmers, being a Civil Servant emerged as a significant factor (*P* = 0.027). Other significant factors included being Female (*P* < 0.001), age over 75 years (*P* < 0.001), Smoking (*P* < 0.001), Alchol (*P* < 0.001), KPS ≤ 80 (*P* = 0.001), tumor grade G3-G4 (*P* < 0.001), lymphovascular invasion (*P* < 0.001), nerve invasion (*P* < 0.001), pathological T stages pT3 (*P* < 0.001) and pT4 (*P* < 0.001), pathological N stages pN1 (*P* < 0.001), pN2 (*P* < 0.001), and pN3 (*P* < 0.001), as well as pathological stages pIII (*P* < 0.001) and pIV (*P* < 0.001). The type of thoracic surgery (*P* = 0.009) and the type of abdominal surgery (OE) (*P* = 0.029) were also significant. Further multivariate analysis revealed that several of these factors remained significant. Specifically, being alchol (*P* < 0.001), age over 75 years (*P* < 0.001), tumor grade G3-G4 (*P* = 0.008), nerve invasion (*P* = 0.035), pN2 (*P* = 0.019),pathological stage pIII (*P* = 0.042) and pIV (*P* = 0.014) were independently associated with survival outcomes (Fig. 5).

## Discussion

This study revealed significant disparities in long-term OS among ESCC patients based on their occupational backgrounds, particularly between Farmers and Civil Servants. In the overall cohort, Civil Servants exhibited a poorer survival outcome compared to Farmers, with a HR of 1.288 (95% CI: 1.028–1.615; *P* = 0.027). This difference persisted after PSM, suggesting that the observed disparity is not merely a reflection of differences in clinical characteristics such as tumor stage. In the male subgroup, the survival difference became significant only after PSM (HR: 1.432; 95% CI: 1.031–1.989; *P* = 0.032), while in the female subgroup, no significant differences were observed in the Cox regression analysis before or after PSM. However, the RMST analysis painted a more nuanced picture, showing that Civil Servants, especially females, had substantially shorter survival times (Crude RMST: 25.59 months vs. 67.32 months for Farmers), despite the small sample size limiting statistical power in this subgroup.

Farmers, who generally have more physically demanding jobs, might benefit from better physical fitness, which could contribute to improved postoperative recovery and resilience against cancer progression. In contrast, Civil Servants, who may experience higher levels of occupational stress and sedentary lifestyles, could face additional health challenges that adversely affect their cancer prognosis. The use of RMST analysis provided additional insights into the survival differences among occupational groups. The RMST analysis revealed that Civil Servants had the lowest survival curve, reflecting their advanced stage at diagnosis. However, after adjusting for confounding factors, the RMST curves for Civil Servants aligned more closely with those of other groups, suggesting that stage at diagnosis plays a critical role in survival disparities.

Occupational environments likely play a pivotal role in shaping health outcomes. Farmers, who predominantly work outdoors and engage in physical labor, may benefit from higher levels of physical activity, which has been associated with improved cardiovascular health and potentially better cancer outcomes. In contrast, Civil Servants, typically confined to indoor office settings, may lead more sedentary lives, compounded by higher stress levels from administrative or managerial responsibilities. Sedentary behavior and chronic stress are known risk factors for poor health, potentially exacerbating cancer progression through mechanisms such as immune suppression or metabolic dysfunction.

Recent studies have emphasized that social and lifestyle stressors are crucial factors influencing cancer development and progression through their significant impact on both mental and physical health, particularly immune function [5, 13, 17, 18]. Various elements contribute to increased cancer risk, including poor lifestyle habits, environmental exposures, chronic work-related stress, and economic pressures [5, 13, 17, 18]. These factors may explain the observed survival differences between farmers and civil servants following esophagectomy for ESCC. Civil servants typically experience distinct types of social stress, often remaining under sustained pressure for extended periods. Conversely, farmers face different stressors related to physical labor and economic uncertainty. The chronic stress experienced by these occupational groups can differentially affect postoperative recovery and long-term overall survival outcomes. The underlying mechanism involves stress-induced suppression of immune responses, which can facilitate cancer growth and metastasis—particularly relevant in ESCC, where immune function plays a vital role in controlling tumor progression [5, 17–19]. Additionally, occupation-related stress can result in varying levels of motivation to adhere to follow-up care and healthy lifestyle changes post-surgery, thereby influencing recovery and survival outcomes between these populations.

As clinical trials continue to advance and neoadjuvant treatment modalities become more widespread, the long-term OS of patients following esophagectomy is steadily improving. In this era of immunotherapy, it is essential not to overlook the impact of various social factors on survival [7, 9, 20, 21]. Future treatment strategies can more focus on identifying high-risk populations and providing personalized treatment plans tailored to the unique circumstances of each patient. Moreover, recognizing the influence of social factors allows for the development of supportive care programs that can mitigate the adverse effects of stress and socio-economic challenges. These programs may include access to mental health resources, nutrition and lifestyle counseling, and assistance with healthcare accessibility. By addressing these elements, the overall resilience of patients can be bolstered, potentially leading to improved compliance with treatment regimens and better health outcomes.

While this study provides insights into the impact of occupational background on the long-term OS of patients with ESCC following esophagectomy, several limitations must be considered when interpreting the results. Firstly, the retrospective design may introduce biases such as selection and information bias, affecting the accuracy and generalizability of the findings. The categorization of occupations into four broad groups may oversimplify the range of occupational exposures and socioeconomic factors influencing health outcomes, not accounting for variations within each category. We acknowledge that a more detailed distinction among various occupations could enhance the study. However, categorizing all occupations and conducting analyses is challenging, particularly since many occupations have fewer than 10 individuals represented. Such low sample sizes could introduce significant bias into our findings. Therefore, we just analyses the four occupations. Additionally, the study's regional focus on Sichuan, China, may limit the applicability of findings to other areas with different healthcare systems and demographics. Lastly, treatment advancements over the study period (2010–2017) could have influenced OS, and the study may not fully capture the impact of newer treatment modalities. Future research should aim to address these limitations by using prospective designs, expanding occupational categories, and incorporating a broader range of confounding factors, with larger multi-center studies providing more generalizable results.

## Conclusion

This study analyzed the OS of patients after esophagectomy across various occupational groups, revealing significant disparities. Civil Servants exhibited poorer survival outcomes compared to Farmers, even after controlling for confounding factors through PSM.

## Data Availability

The authors declare that they have no known competing financial interests or personal relationships that could have appeared to influence the work reported in this paper. The data are anonymous, and the requirement for informed consent was therefore waived.

## Abbreviations

AJCC: American Joint Committee on Cancer
CI: Confidence interval
CSCO: Chinese Society of Clinical Oncology
ESCC: Esophageal squamous cell carcinoma
HR: Hazard ratio
RMST: Restricted mean survival time
MIE: Minimally invasive esophagectomy
OE: Open esophagectomy
OS: Overall survival
LN: Lymph node
TESCC: Thoracic esophageal squamous cell carcinoma
